# Trunk rotational strength asymmetry in adolescents with idiopathic scoliosis: an observational study

**DOI:** 10.1186/1748-7161-2-9

**Published:** 2007-07-09

**Authors:** Kevin L McIntire, Marc A Asher, Douglas C Burton, Wen Liu

**Affiliations:** 1 Department of Physical Therapy and Rehabilitation Sciences, University of Kansas Medical Center, 3901 Rainbow Boulevard, Kansas City, Kansas 66106, USA; 2 Department of Orthopedic Surgery, University of Kansas Medical Center, 3901 Rainbow Boulevard, Kansas City, Kansas 66106, USA

## Abstract

**Background:**

Recent reports have suggested a rotational strength weakness in rotations to the concave side in patients with idiopathic scoliosis. There have been no studies presenting normative values of female adolescent trunk rotational strength to which a comparison of female adolescents with idiopathic scoliosis could be made. The purpose of this study was to determine trunk rotational strength asymmetry in a group of female adolescents with AIS and a comparison group of healthy female adolescents without scoliosis.

**Methods:**

Twenty-six healthy adolescent females served as the healthy group (HG) (average age 14 years) and fourteen otherwise healthy adolescent females with idiopathic scoliosis served as the idiopathic scoliosis group (ISG) (average age 13.5 years, average Cobb 28°). Participant's isometric trunk rotational strength was measured in five randomly ordered trunk positions: neutral, 18° and 36° of right and left pre-rotation. Rotational strength asymmetry was compared within each group and between the two groups using several different measures.

**Results:**

The HG showed strength asymmetry in the 36° pre-rotated trunk positions when rotating towards the midline (p < 0.05). The ISG showed strength asymmetry when rotating towards the concavity of their primary curve from the neutral position (p < 0.05) and when rotating towards the concavity from the 18° (p < 0.05) and 36° (p < 0.05) concave pre-rotated positions. The ISG is significantly weaker than the HG when rotating away from the midline toward the concave (ISG)-left (HG) side from the concave/left pre-rotated 18° (p < 0.05) and 36° (p < 0.05) positions.

**Conclusion:**

The AIS females were found to be significantly weaker when contracting toward their main curve concavity in the neutral and concave pre-rotated positions compared to contractions toward the convexity. These weaknesses were also demonstrated when compared to the group of healthy female adolescent controls. Possible mechanisms for the strength asymmetry in ISG are discussed.

## Background

Accumulated evidence has shown asymmetry in muscle structure, mass, innervation, and activity level in adolescents with idiopathic scoliosis [1-9]. Recently it has been found that an increased EMG ratio between the convex and concave sides of right thoracic curves at the lower end vertebra is linked to curve progression [10-12]. The asymmetric muscle activity is suggested to be associated with increased axial rotation of the spine, which in turn is associated with Cobb angle progression [12]. Based on these findings, it seems logical that trunk strength asymmetry would be present in patients with idiopathic scoliosis. Two recent studies have examined the trunk rotational strength asymmetry in adolescents with idiopathic scoliosis [13,14]. They reported that patients with idiopathic scoliosis were weak when rotating toward their curve's concave side and suggested a relation between the strength asymmetry and progression of the spinal curvature. However, no statistical analyses of the data and no comparison to healthy subjects were provided.

To our knowledge there have been no studies reporting normative values of trunk rotational strength in adolescent females, to which a comparison of female adolescents with idiopathic scoliosis could be made. There have, however, been many studies measuring isometric trunk rotational strength in adults aimed at gaining better understanding of trunk strength, muscle activity, and potential pathophysiology of low back pain [15-26]. None of these studies have shown a rotational strength asymmetry in the healthy adult population and no conclusive evidence indicates that isometric trunk rotational weakness or asymmetry is prognostic or pathologic for low back pain. It is, also unknown whether a trunk rotational strength asymmetry exists in healthy adolescents.

The purpose of this study was to determine trunk rotational strength asymmetry in a group of female adolescents with AIS and a comparison group of healthy female adolescents without scoliosis. The result of this study may help us to better understand the scoliotic condition and provide insight on the etiology or progressive pathology of AIS. It may also help in the development of future non-operative management approaches for AIS.

## Methods

Twenty-six healthy young adolescent females (average age 14 ± 2 years), the healthy group (HG), and fourteen otherwise healthy adolescent females with idiopathic scoliosis (average age of 13.5 ± 1.7 years), the idiopathic scoliosis group (ISG), were recruited into this study. The HG participants responded to flyers posted at the University of Kansas Medical Center and the ISG participants were enrolled at the pediatric spine clinic at the University of Kansas Medical Center. The HG subjects were screened, using a self reported questionnaire, for any previous or current back injury that required a doctor's visit within a year and any lower limb length discrepancy. Additionally the HG subjects were screened for scoliosis using a scoliometer. The ISG subjects were patients seen in the clinic by one (DCB) of the authors. Inclusion criteria for the ISG subjects were a) diagnosis of idiopathic scoliosis; b) Cobb angle of 20° – 45°; c) Risser sign of III or less; and d) age from 10 through 17 years old. Exclusion criteria include patients with any diagnosable neuromuscular disease or other cause of scoliosis. The study was approved by the Institution Research Board at the University of Kansas Medical Center and all subjects and their parent or guardian signed an assent and consent form.

Subjects' age and maturity rating are presented in Table 1. A modified Pubertal Maturation Observational Scale (PMOS) was used to classify subjects into maturational categories: prepubertal (equivalent to Tanner Stage 1), early pubertal (equivalent to Tanner Stages 2 and 3), or late or postpubertal (equivalent to Tanner Stages 4 and 5) [27]. The checklist items were collected using a participant intake questionnaire and investigator observations. The items are based on several indicators of pubertal maturation i.e. the growth spurt, menarche status, breast development, calf muscle definition, and leg and arm pit hair (or have begun to shave legs and arm pits). A numbering system was used (1–3) to represent the pre, early, and late (or post) puberty levels, respectively. The anthropometric measurements of each subject included the weight, height, body fat percentage, and lean body weight (LBW) (Table 1). The body fat percentage and LBW were determined using a validated method [28]. Briefly, this method involves measurements of circumferences (forearm, waist, and hips) and bone diameters at a joint (wrist). From these measurements, calculations are made to compute the lean body weight. Each individual's somatotype (endo, meso, and ectomorphic) was calculated using the Heath-Carter anthropometric somatotyping method [29,30]. Comparisons between groups were made using independent t-tests (Table 1). Information about vigorous and moderate physical activity as well as walking for exercise, walking for transportation and time spent while sedentary was collected from all 26 HG subjects and 9 ISG subjects using a 7-day short form International Physical Activity Questionnaire (IPAQ), which has been validated for its use in the adolescent population [31-34] (Table 1). Results of clinical measurements for subjects in the ISG including spinal curve characteristics are presented in Table 2. The curves were classified as thoracic, double thoracic, double, or thoracolumbar/lumbar following Scoliosis Research Society criteria [35]. We chose the main curve as the thoracic or thoracolumbar/lumbar curve associated with the larger clinical angle of trunk inclination [36] and x-ray angle of vertebral rotation (AVR) [37]

**Table 1:** Anthropometric measurements

	**HG**	**ISG**	
	**Mean(std)**	**Mean(std)**	**p-value**
	**Anthropometric and Somatotpye**		
**Number in Group**	26	14	
**Age (yrs)**	14.04(2.01)	13.51(1.36)	0.20
**Maturity**	2.69(0.55)	2.57(0.51)	0.25
**Height (cm)**	161.17(7.95)	158.66(7.31)	0.17
**Weight [56]**	53.16(14.50)	46.71(9.13)	0.07
**LBW [56]**	41.75(8.13)	39.31(5.26)	0.16
**Body Fat (%)**	20.16(5.83)	14.76(6.20)	**0.01**
**BMI**	20.25(4.01)	18.46(2.97)	0.08
**Endomorph**	4.53(1.98)	3.04(1.12)	**0.01**
**Mesomorph**	3.28(1.28)	3.01(1.04)	0.28
**Ectomorph**	3.22(1.44)	3.89(1.72)	0.11
	**International Physical Activity Questionnaire**		
**Number in Group**	26	9	
**Minutes spent performing:**	**Mean**	**Mean**	**p-value**
**Vigorous**	282.7	333.3	0.61
**Moderate**	143.8	98.3	0.34
**Walking for exercise**	97.5	16.7	**0.02**
**Sedentary**	776.5	746.7	0.74
**Riding in a car**	277.3	272.2	0.93
**Walking for transportation**	84.6	23.3	0.07

Subjects' age, maturity, results of anthropometric measurements, and reported activity levels (average (SD)). P-values of t-test between the HG and ISG are presented. LBW – Lean Body Weight; BMI – Body Mass Index; Endomorph, Mesomorph, Ectomorph – somatotype scores calculated from the Heath-Carter method.

**Table 2:** Curve characteristics for each individual in the ISG

**Accession #**	**Age**	**Menarche**	**Risser**	**TRC**	**HT°**	**T°**	**TL/L°**	**Curve Pattern**	**MainT, TL/L Curve Direction**
**3001**	16.1	no	0	CL	-	37	-	Thoracic	c
**3010**	13.4	yes	III	CL	-	-	21	TL/L	c
**3012**	12.3	no	0	OP	-	-19	-	Thoracic	R
**3013**	12.5	yes	0	CL	37	-35	19	Dbl. Thor.	R
**3014**	10.8	no	0	OP	-	-	20	TL/L	c
**3016**	11.9	no	0	OP	27	-30	-	Dbl. Thor	R
**3017**	15.8	no	III	CL	-	-32	32	Dbl	R
**3018**	14.4	no	0	CL	28	-23	-	Dbl. Thor	R
**3020**	13.2	no	0	CL	-	-24	-	Thoracic	R
**3021**	14.6	yes	I	CL	-	-36	22	Thoracic	R
**3022**	11.7	no	0	OP	21	-14	-	Dbl. Thor	R
**3024**	14.3	yes	III	CL	37	-41	24	Thoracic	R
**3025**	12.0	no	0	CL	-	-20	18	Thoracic	R
**3026**	15.7	no	Nav	CL	-	-	-28	TL/L	R

Age (yrs + mo), Menarchal status, Risser sign, tri-radiate cartilage (TRC), high thoracic Cobb (HT°), thoracic Cobb (T°), thoracolumbar/lumbar Cobb (TL/L°), curve pattern, and largest thoracic or thoracolumbar/lumber curve side for the idiopathic scoliosis group. (Nav – Not Available).

**Figure 1:**
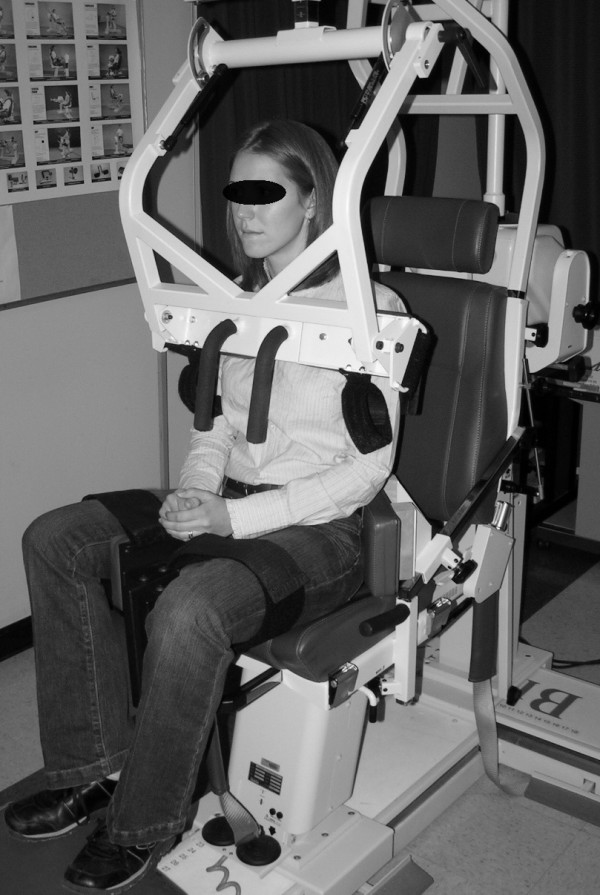
Picture of the testing device.

**Figure 2:**
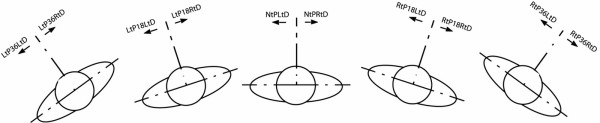
**The five testing positions**. A drawing of the five testing positions along with the two contraction directions. Should be viewed with Table 3.

Isometric trunk rotational strength was measured at five randomly assigned positions of trunk rotation 36°, 18°, 0°, -18°, -36°, with negative values indicating a right pre-rotated position [13,14,38]. The testing protocol is fully described and is reliable [39]. Briefly, a Biodex Multi-joint System 3 Pro (Biodex Medical Systems; Shirley, NY) with a Biodex trunk rotation attachment was used to measure isometric trunk rotational strength. Prior to testing, each subject walked on a treadmill (3.0 mph, 1% grade) for seven minutes to warm up. Once seated in the testing machine the subject's legs and hips were secured using pads and Velcro straps. Their shoulders were secured to the rotational attachment using a Velcro strap (Figure 1). The subject was then randomly positioned in one of the five pre-rotated trunk positions. Neutral (0°) is defined as the shoulders being in line with the pelvis. At each position the subject exerted an isometric rotational contraction in the right direction for five seconds, rested for five to ten seconds, and then in the left direction for five seconds. The contraction sequence was repeated two more times for a total of six alternating contractions (three to the right and three to the left). This procedure was repeated at each of the five trunk positions. Figure 2 shows the five trunk positions and the directions of contractions.

A one second moving average window was used to identify the highest mean torque value with the lowest variation for each isometric contraction; termed *stable one *[39]. The final torque value in a specific contraction direction at each trunk position was an average of all three torques measured in three trials. If there was a 20% difference between any of the three torque values for a particular trial, the lower value was discarded as an inconsistent effort. Torque values were then normalized to lean body weight for further analysis. Strength asymmetry for subjects in the HG was examined between the right and left contraction directions. ISG individuals' torque values and strength asymmetry were assessed in relationship to the main thoracic or thoracolumbar/lumbar curve direction (i.e. convexity or concavity). The measurement of trunk rotational strength and its normalized values have been shown highly reliable [39].

Several studies have shown that from a pre-rotated position away from neutral (0°) isometric trunk rotational strength in a rotation towards the midline was greater than that away from the midline [15,16,23,40-43]. We therefore have termed a rotation towards the midline from a pre-rotated trunk position the *high force arc*, and a rotation away from the midline the *low force arc*. For an isometric contraction at a given pre-rotated trunk position, the contraction towards the midline is termed *high force contraction*, and *low force contraction *if rotating away from the midline. A contraction at the neutral position is termed either *left contraction *or *right contraction *for subjects in HG, and *concave contraction *or *convex contraction *for subjects in ISG.

Within group strength asymmetry was first analyzed using an omnibus F-test on the difference between two sides, i.e. left and right in HG or concave and convex in ISG. This preliminary omnibus test is used to test the hypothesis that the mean difference between the two sides equals zero. This type of statistic was used to reduce the risk of type I error when multiple t-tests were required to compare two sides for multiple pre-rotated trunk position and two contraction directions. If significance was reached, then paired t-tests were used for post-hoc analysis. For differences between groups a MANOVA was used with contractions in both directions serving as two dependent variables and group as the independent variable. Independent t-tests were used for post-hoc analysis. The HG left directional values were compared with the ISG concave values because most of the ISG patients had a right sided main curve (11 of 14). The reciprocal values for the 3 left apex curves in the ISG were used in the data analysis.

Our previous study found that the opposing muscle strength ratio was moderately repeatable and displays a consistent *bowl shaped *pattern [39]. This is the ratio between the measured trunk strength rotating towards the midline (high force contraction) and the trunk strength rotating away from the midline (low force contraction) while in the same pre-rotated trunk position. The ratio at the neutral position was defined as the left contraction divided by the right contraction in HG, and between the concave contraction and convex direction in the ISG. Paired t-tests were used to compare the opposing muscle strength ratios at 18° and 36° positions within each group. An ANOVA was used to analyze opposing muscle ratios between groups. Independent t-tests were used in post-hoc analyses of within group and between group comparisons, respectively.

One additional measure for a side asymmetry was the directional percent side difference. The directional percent side difference is calculated by subtracting the right/convex contraction (which has a negative sign) from the left/concave contraction (in mirrored trunk positions), then dividing the difference from the mean of the two values, and presented as a percentage value. A positive directional difference would indicate a weakness in rotating towards the right/convex side and a negative value would indicate a weakness towards the left/concave side. An F-test was used to test the hypothesis that within a group the means of percent differences at each trunk position were equal to zero. An ANOVA was used to analyze directional percent side differences between groups. Independent t-tests were used in post-hoc analyses of between group comparisons. The statistical software SPSS 11.0 (SPSS Inc. Chicago, Ill) was used for all analysis with an alpha level at p < 0.05.

## Results

### Within Group Symmetry Differences

**Figure 3:**
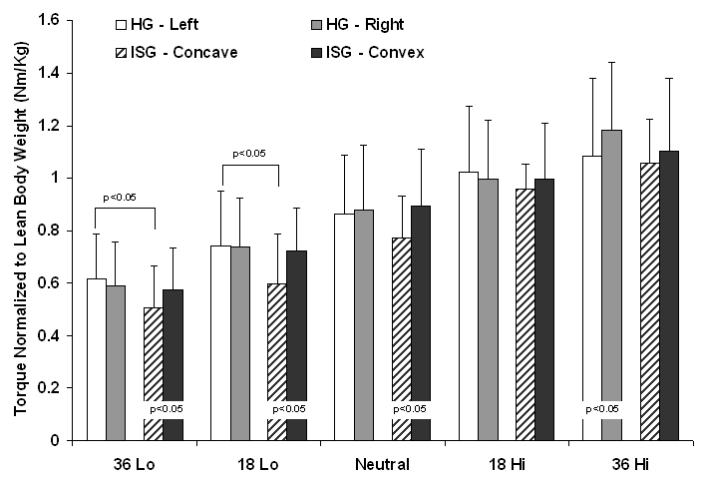
**Normalized torque values for the control group (HG) and the idiopathic scoliosis group (ISG)**. Torque normalized to lean body weight for the control group (HG, n = 26) and the idiopathic scoliosis group (ISG, n = 14). Significant p-values for within group differences are presented at the base of the graph. Significant p-values for between group differences are shown above the values with brackets indicating which values were analyzed. Specifically HG left compared to ISG concave in the 36 Low force and 18 Low force contractions.

Normalized trunk rotational strength values and standard deviations are presented in Figure 3 and Table 3. The initial omnibus test for differences between the two sides was significant (p < 0.01). Subsequent paired t-tests for HG revealed a significant weakness in the right 36° high force contraction (1.08 ± 0.3 Nm/Kg) compared to the left 36° high force contraction (1.18 ± 0.26 Nm/Kg) (p < 0.05). For the ISG there were significant weaknesses at the neutral position when rotating towards the concavity compared to rotating towards the convexity (0.77 ± 0.16 Nm/Kg versus 0.89 ± 0.22 Nm/Kg, p < 0.05), at concave 18° side low force contraction (0.59 ± 0.19 Nm/Kg versus 0.72 ± 0.16 Nm/Kg, p < 0.05) and concave 36° low force contraction (0.50 ± 0.16 Nm/Kg versus 0.57 ± 0.16 Nm/Kg, p < 0.05).

**Table 3:** Isometric trunk rotational strength values

	**36°**		**18°**		**Neutral**		**-18°**		**-36°**	
**Pre-Rotation Side**	**Left/Concave**					**Right/Convex**				
**Contraction Direction**	Left/Concave	Right/Convex	Left/Concave	Right/Convex	Left/Concave	Right/Convex	Left/Concave	Right/Convex	Left/Concave	Right/Convex

**Force Arc**	Low	High	Low	High			High	Low	High	Low

	**Absolute Values**									
**HG (n = 24)**	25.8(8.2)	49.7(13.5)	31.2(9.7)	41.4(10.7)	35.9(10.1)	36.7(11.1)	42.9(12.7)	31.1(10)	44.9(12.6)	24.7(7.8)
**ISG (n = 14)**	19.6(6.9)	42.5(11.8)	24.0(7.5)	38.7(11.6)	30.5(6.8)	35.2(9.8)	38.2(4.6)	28.3(8.7)	41.9(7.4)	22.9(7.2)
**p-values <**	**0.05**	ns	**0.05**	ns	**0.05**	ns	ns	ns	ns	ns

	**Normalized to Lean Body Weight**									
**HG (n = 24)**	0.62(.17)	1.18(.26)*	0.74(.21)	1.00(.22)	0.86(.22)	0.88(.25)	1.02(.25)	0.74(.19)	1.08(.30)*	0.59(.17)
**ISG (n = 14)**	0.50(.16)**†††**	1.10(.27)	0.59(.19)**††**	0.99(.22)	0.77(.16)**†**	0.89(.22)†	0.96(.10)	0.72(.16)**††**	1.05(.17)	0.57(.16)**†††**
**p-values <**	**0.05**	ns	**0.01**	ns	ns	ns	ns	ns	ns	ns

Trunk rotational strength presented in absolute values and normalized to Lean Body Weight (Nm/Kg) (with standard deviations) measured at five trunk positions in two contraction directions. If viewed with Figure 2 it illustrates the five trunk positions, i.e. rotated to the left 36° or 18°, neutral position, and rotated to the right 18°, or 36°. Strength values in two contraction directions, left or right for the healthy group (HG) and concave or convex for the idiopathic scoliosis group (ISG), are shown below each position figure. P-values are listed directly below the vales compared between the HG and ISG. Within group significant differences: *, †, ††, ††† = p < 0.05

### Between Group Differences

The MANOVA showed a significant group difference in trunk strength towards the left/concave direction (p < 0.05). Independent t-tests revealed the ISG to be significantly weaker than the HG in the two low force contraction pre-positions: ISG concave 36° versus HG left 36° (0.50 ± 0.16 Nm/Kg compared to 0.62 ± 0.17 Nm/Kg, p < 0.05) and ISG concave 18° versus HG left 18° (0.59 ± 0.19 Nm/Kg versus 0.74 ± 0.21 Nm/Kg, p < 0.05).

### Opposing Muscle Ratio

**Figure 4:**
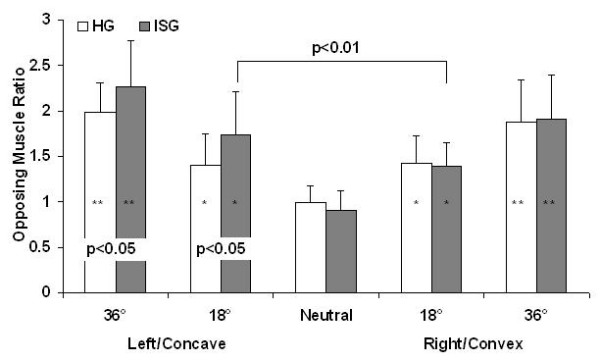
**Opposing muscle ratios**. Opposing muscle ratio. This is the ratio between the measured trunk strength rotating towards the midline (high force contraction) and the trunk strength rotating away from the midline (low force contraction) while in the same pre-rotated trunk position. Ratios in the neutral position for both HG (n = 26) and ISG (n = 14) were significantly lower than all other values (p < 0.001). Eighteen degree (18°) ratios for the ISG were significantly lower than the same side 36° ratios (p < 0.01). P-values for between group differences are presented at the base of the graph. The significant side difference is presented using a bracket to identify the values used, with the p-value shown on top. [* Significantly higher than the neutral position; ** significantly higher than the neutral and 18° positions (on the same side)]

For the HG group independent t-tests with a Bonferroni correction showed no differences between the right 18° ratio and left 18° ratio or between the right 36° ratio and the left 36° ratio (Figure 4). A significant difference was shown in the ISG between the concave 18° ratio and the convex 18° ratio (1.74 ± 0.47 versus 1.39 ± 0.26 respectively) (p < 0.01) (Figure 4). The ANOVA for opposing muscle ratio revealed that the variances for the groups were not equal. Therefore, the data was transformed to achieve equal variances using a power transform calculation function contained in the SPSS program. The ANOVA performed on the transformed data showed a significant difference for position (p < 0.001) and an interaction between group and position (p < 0.05). Post-hoc analysis showed that for both HG and ISG the ratio differences were significantly lower in the neutral position than all other ratios (p < 0.001). For the HG the right 18° ratio (1.42 ± 0.3) was significantly lower than the right 36° ratio (1.88 ± 0.46) (p < 0.001). The same was true on the left side for the 18° (1.41 ± 0.34) and 36° (1.98 ± 0.33) ratios (p < 0.001). For the ISG the convex 18° ratio (1.39 ± 0.26) was significantly lower than the convex 36° ratio (1.92 ± 0.47) (p < 0.01). The same was true for the concave 18° (1.74 ± 0.48) and 36° (2.27 ± 0.5) ratios (p < 0.01). Between group differences showed a significant difference between the concave/left 36° and 18° ratios on the side (p < 0.05).

### Directional Percent Side Difference

**Figure 5:**
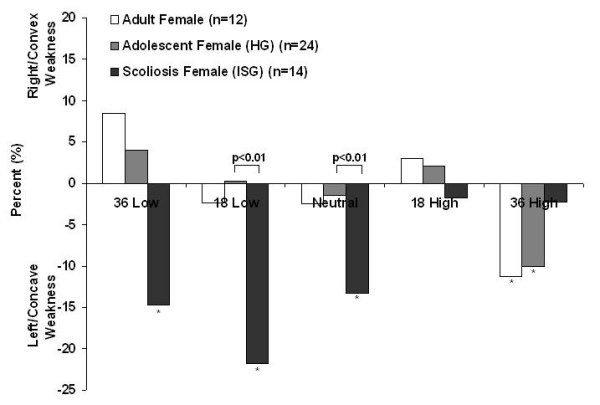
**Directional percent side differences**. Directional percent side differences for adult females taken from McIntire et al (2007) and for both groups from the current study. The adult female values were included to display the similar pattern seen from the HG. (See text for standard deviations). [* Indicates significantly different than zero (p < 0.05). Brackets and p-values are shown for HG versus ISG comparisons].

The F-test showed that mean percent side difference at 36 High (-10 ± 22%) for the HG was significantly (p < 0.05) different from zero. For the ISG mean percent side difference were significantly different from zero at 36 Low (-15 ± 29%), 18 Low (-21 ± 32%) and neutral (-13 ± 22%) (p < 0.05, p < 0.001, and p < 0.05 respectively) (Figure 5). The differences between HG and ISG were significant at 36 Low (4 ± 18% versus -15 ± 29%, p < 0.01), 18 Low (0 ± 21% versus -21 ± 32%, p < 0.01) and neutral (-1.5 ± 18% versus -13 ± 23%, p < 0.05). The directional percent side difference of a group of healthy female adults from our previous work [39] had a similar pattern as the HG (Figure 5).

## Discussion

To our knowledge, there have been no previous studies of trunk rotational strength asymmetry in a group of healthy adolescent females. Two recent studies reported individual trunk rotational strength asymmetry in patients with idiopathic scoliosis, but provided no group statistics or normative value for comparisons [13,14]. There have been a number of studies that reported trunk rotational strength in the healthy adults and patients with low back pain [16-21,23-26]. Recently, we have observed a side difference in healthy male and female adults in a high force contraction at a 36° pre-rotated position [39]. Similarly in the current study, healthy female adolescents showed a significant weakness at the right 36° high force contraction compared to the left 36° high force contraction. In contrast, female adolescents with idiopathic scoliosis in the current study showed significant weakness in the neutral position and the two pre-rotated positions to the concavity in comparison to the mirror trunk position. Although the strength asymmetry could be the result of either weaker concave side or stronger convex side, the comparison between healthy female adolescents and the scoliotic patients showed weakness associated with idiopathic scoliosis in low force contractions to the concave/left sides, but no difference between two groups to the convex/right sides.

The measure of opposing muscle ratio showed also trunk strength asymmetry. The bell shaped pattern of the five ratios in the HG was similar to the pattern observed in healthy adults in our previous study [39]. The ratio in the ISG was greater at the 18° concave side than 18° convex ratio, which in this case is a reflection of the weaker low force contraction, i.e. the ratios denominator. Furthermore, differences between the healthy and patient groups showed significance between the left (HG) and concave (ISG) ratios at both 36° and 18° trunk positions. These results further confirmed the strength asymmetry in the patients with idiopathic scoliosis.

Two healthy groups, i.e. healthy female adolescents in the current study and healthy female adults in our previous study [39], showed similar patterns in the directional percent side difference with maximum mean differences from zero of about 10%, significant only at the 36° high force contraction for the healthy adolescent females (HG). However, in the ISG the percent side differences were significant at both of the low force contractions as well as at the neutral position; and the maximum mean differences were as high as 27%. The difference between the two healthy groups and the idiopathic scoliosis group in directional percent side difference showed clearly different patterns (Figure 5). This measure may become useful as an alternative way of define side asymmetry in trunk strength.

The major finding of asymmetric trunk strength in the current study was generally in agreement with two past studies that reported a strength asymmetry weakness when rotating in the concave direction [13,14]. Since neither strength values nor statistical results were presented in their reports a direct comparison between the current study and their studies was not possible. However, one noted difference was the reported positions of trunk weakness. They reported trunk strength asymmetry at all five trunk positions, with weakness when rotating towards the concavity, ranging from 12% to 47% whereas the current study found significant weakness only in the concave 36°, 18°, and neutral positions when contracting towards the concavity, individual values ranging from 0% to 80%.

Multiple factors might be responsible for the measured trunk strength asymmetry in female adolescents with scoliosis. Past studies have reported differences in cross sectional area, fiber type, and activation level between normal and AIS paraspinal muscles [4-6,9,44-50]. All these factors may influence force generation capacity of the muscle [20,51-54]. Multiple muscle groups are involved in rotating the trunk. Among the most important muscle groups for trunk rotation are the oblique abdominal muscles [25,43,48]. It is possible that the measured strength asymmetry is a result of altered biomechanics of the oblique abdominal muscles due to the asymmetrical torso. Mooney et al. [14] suggested that the trunk strength weakness was due to the muscle inhibition of the paraspinal muscles based on their EMG data of the lumbar paraspinal muscles. Trunk paraspinal musculature has been estimated to contribute about 5% of the total torque involved in trunk rotation [53]. The asymmetrical differences in trunk strength found in the current study, ranging from 2 Nm to 5 Nm (absolute torque in Table 3), might be partially due to paraspinal muscle weakness given their suggested 5% contribution.

Other factors for the strength asymmetry might include soft tissue and/or bony deformations, apical vertebral rotation, or range of motion of the participant in the axial plane. Torque values for contractions away from the neutral position (low force) are lower than torque values for contractions toward the neutral position (high force) [14-16,18,22,40-42,55]. Muscle geometry, antagonistic muscles, and soft tissue such as vertebral discs or ligaments have also been suggested for this phenomenon [16,41,42,55]. Patients with scoliosis can have stiffer spines, altered muscle geometry, and vertebral disc and ligament deformity. Those may also affect the measured trunk strength and asymmetry. Baseline apical vertebral rotation was available for eleven of the ISG participants and no correlation was found between the amount of rotation and any measure of strength asymmetry. The AVR for most curves were small (average 10° range 0 – 20°) and it is possible that with a larger AVR, as seen in more advanced curves, that the AVR would show more influence. Range of motion was not measured for any study participant. However, none of the participants had any restrictions in any of the five testing positions. Any specific above mentioned factor could become an important topic for future studies.

Our study has several limitations. The relatively small number of participants, especially in the ISG, and non-randomized sampling process may limit the generalization of results. Several paired t-tests were used in analyzing trunk rotational strength side differences without a Bonferroni correction for reducing type I errors. We believed that the type I error in our results was limited since we used first an omnibus F-test to confirm the presence of side differences in the data. Further more, the side differences in the low force contraction were consistent in the neutral and two concave trunk positions, i.e. the full low force arc. The other limitation is that two groups (HG and ISG) were not specifically matched for age and pubertal status. However, there were no significant differences between two groups in terms of age, pubertal status, as well as their activity level or the time spent while sedentary.

## Conclusion

This preliminary study measured trunk rotational strength in a group of adolescent healthy females and a group of adolescent idiopathic scoliosis females. Scoliosis patients were significantly weaker when rotating towards the concavity of the spinal curve in the neutral position and when pre-rotated 18° and 36° toward the concavity and then contracting towards the concavity, i.e. away from neutral, termed "low force". In contrast, the healthy group did not show weakness in the low force arc. In addition, low force arc trunk strengths on the concave side in scoliotic individuals were also significantly lower than those on the left side in the healthy subjects. These finding may help future researchers develop effective new approaches for the management of idiopathic scoliosis.

## Competing interests

Although not a competing interest for this study, MA declares a 30% ownership of Isola Implants Inc. and DB is a consultant for DePuy Spine.

The other authors declare that they have no competing interests

## Authors' contributions

KM strength tested all study participants, performed statistical analysis, and wrote several sections. MA compiled all clinical data, wrote several sections and edited. DB recruited clinical subjects and edited. WL helped with study design, wrote several sections, edited, and assisted with statistical analysis. All authors read and approved the final manuscript.
